# Association mapping of local adaptation traits of Scots pine in a European wide population sample

**DOI:** 10.1186/1753-6561-5-S7-P38

**Published:** 2011-09-13

**Authors:** Timo Knürr, Sonja Kujala, Mikko J Sillanpää, Komlan Avia, Aleksia Vaattovaara, Katri Kärkkäinen, Outi Savolainen

**Affiliations:** 1 Dept. Mathematics and Stastistics, University of Helsinki, Finland; 2 Dept. biology, Unviersity of Oulu, Finland; 3 Dept. Mathematics and Statistics, University of Helsinki, Finland; 4 Dept. Biology, University of Oulu, Finland; 5 Finnish Forest Research Institute, Finland

## 

Traits related to local adaptation by definition show high phenotypic differentiation. The underlying genetic patterns could be clines at individual loci or small effects and extensive linkage disequilibrium at the underlying loci. In any case, including many populations in an analysis provides more information, but may simultaneously induce problems due to genetic structure. Even if the neutral loci have little genetic structure, loci related to other clinally selected traits could show more structure. Here we have developed an approach to efficiently use the information along a latitudinal environmental gradient. Scots pine populations from central Europe to the species' northern range were sampled and patterns of phenotypic variation of both timing of budset and frost tolerance were measured in common garden experiments, (10 populations, a total of 270 halfsib families, 25 trees per family). By hierarchical modelling of the phenotype's clinal variation and accounting for varying allele frequencies across the 10 populations, the statistical approach simultaneously exploits the genetic variation between and within populations to detect association signals. We apply shrinkage-based Bayesian variable selection to detect genetic associations between timing of bud set and ~450 SNPs in Scots pine.

